# Real-world optimization of tunnel lengths in tunneled peripherally inserted central catheters for cancer patients: A multi-center retrospective cohort study

**DOI:** 10.1371/journal.pone.0338692

**Published:** 2025-12-17

**Authors:** Yinyin Wu, Wei Ding, Yuying Liu, Qianhong Deng, Fengqin Tao, Hanbin Chen, Chang Chen, Meng Xiao, Bilong Feng

**Affiliations:** 1 Department of Nursing, Zhongnan Hospital of Wuhan University, Wuhan, Hubei, China; 2 Department of Lung Oncology and Chemoradiotherapy, Zhongnan Hospital of Wuhan University, Wuhan, Hubei, China; 3 Intravenous Therapy Clinic, Zhongnan Hospital of Wuhan University, Wuhan, Hubei, China; 4 Department of Gastrointestinal Surgery, Zhongnan Hospital of Wuhan University, Wuhan, Hubei, China; 5 Department of Head, Neck and Pediatric Oncology, Zhongnan Hospital of Wuhan University, Wuhan, Hubei, China; 6 Department of Intensive Care Unit, Zhongnan Hospital of Wuhan University, Wuhan, Hubei, China; Baylor College of Medicine, UNITED STATES OF AMERICA

## Abstract

**Background:**

Standardized guidelines for optimal tunnel length in tunneled peripherally inserted central catheters (PICCs) are lacking.

**Objectives:**

The objective of this study was to evaluate the real-world impact of tunnel length on clinical outcomes.

**Methods:**

This retrospective cohort study included 207 cancer patients who received tunneled PICCs, categorized into a control group (tunnel length > 4 cm, n = 134) and an observation group (tunnel length ≤ 4 cm, n = 73). Propensity score matching (PSM) was used to address baseline heterogeneity. Cox regression analyses were used to assess the risk of complication during a 120-day follow-up.

**Results:**

Compared to the control group (tunnel length > 4 cm), the observation group (tunnel length ≤ 4 cm) had a significantly higher adjusted overall complication risk (HR = 2.92, 95% CI: 1.07–7.94, P = 0.036) and unplanned catheter removal rate (4.4% vs. 0.0%, P = 0.027), confirming the safety of longer tunnels despite comparable comfort levels between groups. After PSM, Cox regression analysis showed results consistent with those from the unmatched cohort. Subgroup analyses revealed a reduced risk of complications with longer tunnels in patients with BMI ≤ 25 kg/m² (HR = 0.29, 95% CI: 0.11–0.82), without hypertension (HR = 0.36, 95% CI: 0.13–1.00), without diabetes (HR = 0.38, 95% CI: 0.15–0.97), and with solid tumors (HR = 0.31, 95% CI: 0.11–0.85).

**Conclusion:**

The results show that tunnel lengths > 4 cm reduce overall complications and prolong catheter retention, supporting the implementation of standardized protocols while advocating for personalized adjustments based on BMI, comorbidities, and cancer type.

## Introduction

Peripherally inserted central catheters (PICCs) are increasingly preferred for cancer patients requiring long-term chemotherapy to mitigate the risk of tissue necrosis caused by drug extravasation [1]. However, PICC-related complications remain prevalent. A Chinese multi-center study across 17 hospitals reported a 2.9%−45.5% incidence of catheter-related complications among cancer patients in 2022, encompassing catheter-related thrombosis, phlebitis, infection, drainage, and catheter occlusion [2]. These complications not only compromise patient comfort [3] but also increase medical costs, impair treatment efficacy, and increase the unplanned catheter removal rate [4]. Additionally, the American Infusion Nursing Society recommends a catheter-to-vein ratio of ≤ 45%, posing challenges for patients with small vessel diameters or vascular malformations [5].

The rapid advancement of medical technology has brought subcutaneous tunneling technology into focus as a method for constructing vascular access. Subcutaneous tunneling involves placing a catheter beneath the skin [6] and has been successfully integrated into PICC placement, with multiple randomized controlled trials demonstrating its ability to improve patient outcomes [7,8]. Tunneled PICC extends conventional PICC insertion by changing the vessel access site to the high upper third of the arm, while the catheter exit site is positioned in a safe location (the middle one-third of the upper arm) by creating a subcutaneous tunnel [9]. Its synergy with the Zone Insertion Method (ZIM) is advocated as optimal practice [10,11], aiming for catheter placement in the upper arm’s upper third to achieve a catheter-to-vein ratio of ≤ 45% [5,11]. This strategy enhances venous blood flow, reduces the risk of catheter-related thrombosis, and optimizes exit site position, benefiting patients with small vessel diameters or vascular malformations [12,13].

The latest meta-analysis confirms that the brachial tunneled PICC technique is safe and significantly reduces the risk of late complications compared with conventional PICCs[12]. In tunneled PICC, the tunnel length is defined as the straight distance between the catheter insertion and exit points, measured using a sterile ruler. However, current clinical practice lacks standardized guidelines for tunnel length, with reported ranges varying widely (2–10 cm) [14]. This lack of evidence-based consensus leads to inconsistencies in clinical application. A recent single-center randomized controlled study associated tunnel lengths ≥ 4 cm with a lower risk of complications [15], but real-world optimization (adjusting tunnel length for practical clinical scenarios, e.g., patient heterogeneity, multi-center variations) remains unclear due to the heterogeneity of patient characteristics.

Excessively short tunnels may fail to leverage tunneling benefits, while excessively long lengths increase insertion difficulty and reduce comfort [15]. Previous studies have primarily focused on idealized scenarios through single-center randomized controlled trials, limiting their generalizability. Despite advancements in tunneling techniques, the absence of standardized guidelines for tunnel length necessitates evidence-based recommendations to optimize clinical outcomes. To address this gap, this multicenter retrospective cohort study evaluates the real-world impact of tunnel length on long-term catheter retention by comparing the control group (tunnel length > 4 cm) and the observation group (tunnel length ≤ 4 cm).

## Methods

### Study design, participants, and setting

This multi-center retrospective cohort study analyzed secondary data from a multi-center RCT (NCT05621473) from 12 hospitals in Hubei Province. The sample size in multi-center RCT was calculated based on previous systematic review data [12], with a target of ≥ 206 patients per group (α = 0.05, power = 0.90, 15% dropout). All cancer patients aged ≥ 18 years who underwent tunneled PICC placement between January and October 2023 were eligible if they were willing and able to comply with follow-up. Exclusion criteria included (1) any contraindications to PICC placement: allergy to catheter materials, prospective insertion site infection, local injuries, past irradiation at the insertion site, venous thrombosis, vascular surgery, axillary lymph node dissection, or superior vena cava compression syndrome; (2) lost to follow-up; and (3) missing critical data. The data used in this study were accessed on December 1, 2023 for research purposes. This retrospective cohort study was reported in accordance with the STROBE guidelines. Ethical approval was obtained from the participating hospitals (Reference Number: 2022126). Written informed consent was obtained from all participants, and all data were collected and stored in an anonymized format.

### Catheter placement and maintenance

Catheter placement was performed by a fixed team of PICC specialist nurses with more than 5 years of experience, who had undergone uniform training and obtained certification from the leading central hospital. All procedures were conducted under electrocardiography(ECG) guidance with Doppler ultrasound. Before tourniquet application, B-mode ultrasound was used to assess the vascular status of the upper arm and mark the catheter exit point. A 65-cm single-lumen uncuffed PICC with a tail-cut Seldinger-type 4Fr valve (Branden Medical Devices Co., Shandong, China) was inserted under local anesthesia at both the puncture and exit sites.

Once the catheter entered the vessel, local anesthesia and skin dilation were performed successively at the venipuncture site and the tunnel entrance. Then, normal saline was used to dilate the subcutaneous tunnel, and a tunnel needle (Branden Medical Devices Co., Shandong, China) was inserted to create a suitable pathway from the venipuncture site to the catheter exit point. The catheter exit point and puncture site were determined according to the Zone Insertion Method^TM^ [16], with the puncture site located in the yellow zone and the exit point in the green zone. The catheter tip was positioned at the cavo-atrial junction (CAJ) using intracardiac electrocardiogram(IC-ECG) positioning, and the catheter length was trimmed to maintain an exposed length of 5–7 cm. The wound was closed with octyl cyanoacrylate skin adhesive (Compont Medical Equipment Co., Beijing, China), and the catheter was secured with a sterile transparent waterproof dressing. It should be noted that semipermeable membranes were not used in this study.

PICC maintenance included regular dressing changes, catheter flushing and locking, and complication management. Skin disinfection was performed using 75% ethanol followed by 1% povidone-iodine at the puncture and exit sites. Dressings were replaced 48 hours after catheter placement and weekly thereafter, with immediate replacement if they were contaminated, curled, loose, or wet. Persistent drainage was managed with absorbable gelatin sponges and folded gauze pads. Mechanical phlebitis was treated with warm compresses with hirudoid cream (5–6 times daily). Hypoallergenic dressings were used to prevent medical adhesive-related skin lesions (MARSI). Catheter-related venous thrombosis was managed with low molecular weight heparin and oral warfarin. Central line-associated bloodstream infections (CLABSIs) were treated with antibiotics, and the catheter was removed if necessary. Catheter dislodgement exceeding 5 cm, confirmed by X-ray, led to catheter removal depending on the patient’s treatment condition.

### Data collection

Initial data included demographic (gender, age, BMI, cancer type, comorbidities, history of surgery, smoking, and previous PICC placement), coagulation parameters (platelet count, prothrombin time (PT), activated partial thromboplastin time (APTT), thrombin time (TT), D-dimer), and PICC placement characteristics (insertion arm and vein, arm circumference, one-puncture success, cannulation time(minutes), insertion length, exposure length, and catheter indwelling time(days)). Catheter indwelling time was calculated from the day of catheter insertion to the day of catheter removal. Catheters were removed when (1) the patient’s chemotherapy was completed, or (2) irreversible catheter-related complications occurred (e.g., severe central line-associated bloodstream infection [CLABSI], catheter-related thrombosis unresponsive to anticoagulant therapy) [5]. A fixed PICC specialist nurse conducted follow-up assessments via a WeChat group at 7 ± 3, 30 ± 7, 60 ± 10, 90 ± 10, and 120 ± 10 days after catheterization until treatment completion or catheter removal.

The primary outcome was the incidence of overall complications after catheterization, including: (1) Blood oozing: > 24 hours of bleeding from the puncture point or tunnel exit along the catheter [17];(2) MARSI: Erythema or other cutaneous abnormality (including blisters, erosion, and tears) lasting > 30 minutes after adhesive dressing removal [18]; (3) Local infection: Puncture site infection and tunnel exit infection as defined by the criteria of Infectious Diseases Society of America [19]; (4) Phlebitis: Venipuncture site pain, redness, swelling, induration, purulent exudate, or palpable cord-like veins [20];(5) Catheter-related thrombosis: Thrombus in the catheterization vein confirmed by ultrasonography or CT [21]; (6) Catheter dislodgement:catheter tip displacement of ≥ 5 cm.

The secondary outcomes included patient comfort level and unplanned catheter removal rate. Patient comfort level was self-reported using a 3-point Likert scale (Grade 1: Comfort; Grade 2: Discomfort, tolerable; Grade 3: Discomfort, intolerable). Unplanned catheter removal was defined as any accidental or patient-initiated catheter removal during the medical process [22].

### Statistical analysis

Statistical analyses were performed using SPSS 26.0 (IBM, Armonk, NY, USA) and R version 3.3.3 (R Foundation, Vienna, Austria). Descriptive data were presented as mean and standard deviation (SD), median and interquartile range (IQR), or frequency (n) and percentage (%). Inter-group comparisons of baseline characteristics and secondary outcomes used the Student’s t-test for normally distributed continuous variables, the Mann-Whitney U test for non-normally distributions variables, and the Chi-square or Fisher’s exact test for categorical variables. To address baseline heterogeneity and potential selection bias, propensity score matching (PSM) was implemented using 1:1 nearest-neighbor matching with a caliper of 0.02 logit SD. Propensity scores were derived from logistic regression incorporating variables that were significant at p < 0.10 in univariate analysis.

The Cox proportional hazards regression model was used to analyze the association between tunnel length classification and risk of catheter-related complication in cancer patients. In univariate analysis, each complication type was included as the state variable, catheter indwelling time as the time variable, and tunnel length classification as the independent variable in the model. In multivariate analysis, significant baseline covariates were added as confounding variables to adjust the hazard ratios. Subgroup analyses were performed by age, gender, BMI, PICC placement history, surgical history, coronary heart disease (CHD), diabetes, hypertension, history of CRT, cancer type, coagulation parameters, insertion arm, and insertion vein. All missing data were replaced with the corresponding group means. The statistical significance was defined as P < 0.05.

### Quality control

All researchers underwent uniform training before the trial, covering tunneled PICC placement, complication observation, follow-up procedures, adverse event reporting and handling, and data collection. Enrollment began simultaneously across all centers on January 1, 2023.

## Results

### Participant characteristics

**Fig 1:**
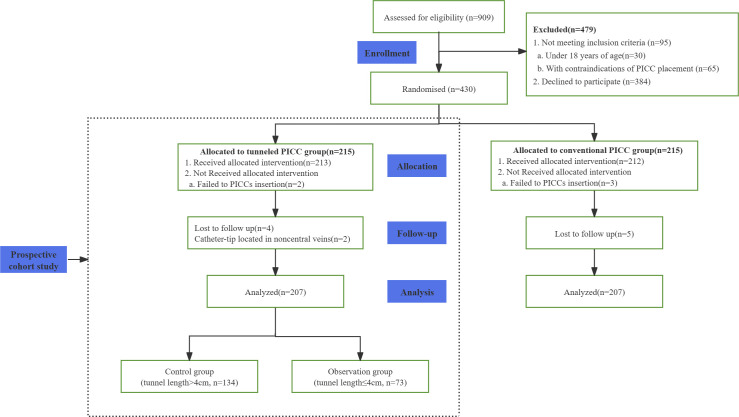
Study outline. PICC, peripherally inserted central catheter.

The participants’ flow chart is detailed in **Fig 1**. Of the 215 participants randomized to the tunneled PICC group, two patients failed PICC insertion and six were excluded after catheterization owing to malpositioned catheter tips (n = 5) or loss to follow-up (n = 1), leaving 207 patients for final analysis (134 in the control group; 73 in the observation group).

**Table 1** presents the participants’ characteristics before and after PSM. The observation group (mean age: 59.11 years) and control group (mean age: 60.75 years) comprised only cancer patients. Among these patients, gastrointestinal malignancies were the most common, followed by breast cancer. Significant differences were observed in cancer type, surgical history, and insertion vein type between the two groups (*P* < 0.05). In addition, the observation group had a significantly shorter catheter indwelling time than the control group (124.0 [68, 151.25] vs. 144.5 [109.75, 183.5], *P* = 0.006). After PSM, the baseline characteristics of the two groups were generally balanced.

**Table 1:** Baseline characteristics of participants before and after PSM (N = 207).

Characteristics	Before PSM			After PSM		
	Control group (n = 134)	Observation group (n = 73)	*P* Value	Control group (n = 57)	Observation group (n = 57)	*P* Value
**Characteristics before catheterization**						
Age, year, mean±SD	59.11 ± 10.66	60.75 ± 12.28	0.338	58.23 ± 11.38	58.88 ± 12.55	0.773
Gender, female, n (%)	74 (55.2)	37 (50.7)	0.562	30 (52.6)	30 (52.6)	1.000
BMI, kg/m^2^, mean±SD	22.74 ± 3.11	22.47 ± 3.08	0.551	22.42 ± 2.93	22.48 ± 3.05	0.913
Cancer type, n (%)			**0.039**			0.356
Head–neck cancer	22 (16.4)	3 (4.1)		8 (14.0)	3 (5.3)	
Breast cancer	16 (11.9)	16 (21.9)		7 (12.3)	14 (24.6)	
Lung cancer	16 (11.9)	11 (15.1)		7 (12.3)	11 (19.3)	
Gastrointestinal cancer	32 (23.9)	25 (34.2)		16 (28.1)	16 (28.1)	
Pelvic cancer	21 (15.7)	7 (9.6)		8 (14.0)	6 (10.5)	
Hematological malignancy	16 (11.9)	6 (8.2)		7 (12.3)	4 (7.0)	
Others	11(8.2)	5 (6.8)		4 (7.0)	3 (5.3)	
Hypertension, yes, n (%)	26 (19.4)	7 (9.6)	0.075	6 (10.5)	6 (10.5)	1.000
Diabetes, yes, n (%)	8 (6.0)	4 (5.5)	1.000	5 (8.8)	4 (7.0)	1.000
CHD, yes, n (%)	1 (0.7)	1 (1.4)	1.000	1 (1.8)	1 (1.8)	1.000
Surgical history, yes, n (%)	69 (51.5)	24 (32.9)	**0.013**	28 (49.1)	23 (40.4)	0.451
PICC placement history, yes, n (%)	10 (7.5)	7 (9.6%)	0.604	5 (8.8)	4 (7.0)	1.000
CRT history, yes, n (%)	1 (0.7%)	1 (1.4%)	1.000	0 (0.0)	1 (1.8)	1.000
Smoke history, yes, n (%)	10 (7.5)	2 (2.7)	0.281	2 (3.5)	2 (3.5)	1.000
PLT (10^9/L), n (%)			0.359			0.739
Low	9 (6.7)	8 (11.0)		5 (8.8)	4 (7.0)	
Normal	114 (85.1)	56 (76.7)		49 (86.0)	47 (82.5)	
High	11 (8.2)	9 (12.3)		3 (5.3)	6 (10.5)	
PT (seconds), n (%)			0.443			0.778
Low	2 (1.5)	3 (4.1)		1 (1.8)	3 (5.3)	
Normal	118 (88.1)	61 (83.6)		48 (84.2)	46 (80.7)	
High	14 (10.4)	9 (12.3)		8 (14.0)	8 (14.0)	
APTT (seconds), n (%)			0.099			0.068
Low	18 (13.4)	5 (6.8)		7 (12.3)	4 (7.0)	
Normal	105 (78.4)	66 (90.4)		42 (73.7)	51 (89.5)	
High	11 (8.2)	2 (2.7)		8 (14.0)	2 (3.5)	
TT (seconds), n (%)			0.476			1.000
Low	4 (3.0)	3 (4.1)		2 (3.5)	1 (1.8)	
Normal	115 (85.8)	58 (79.5)		46 (80.7)	47 (82.5)	
High	15 (11.2)	12 (16.4)		9 (15.8)	9 (15.8)	
D-dimer (ng/ml), positive, n (%)	57 (43.8)	33 (45.8)	0.282	23 (40.4)	27 (47.4)	0.269
Insertion arm, n (%)			0.239			0.454
Left	52 (38.8)	35 (47.9)		31 (54.4)	26 (45.6)	
Right	82 (61.2)	38 (52.1)		26 (45.6)	31 (54.4)	
Insertion vein, n (%)			**0.011**			1.000
Basilic vein	105 (78.4)	68 (93.2)		52 (91.2)	52 (91.2)	
Brachial vein	27 (20.1)	5 (6.8)		5 (8.8)	5 (8.8)	
Cephalic vein	2 (1.5)	0 (0.0)		—	—	
Arm circumference, cm, mean ± SD	27.53 ± 3.39	27.30 ± 3.29	0.646	27.35 ± 3.03	27.47 ± 3.24	0.835
**Characteristics after catheterization**						
One-puncture success, yes, n (%)	131 (97.8)	72 (98.6)	0.664	57 (100)	57 (100)	—
Cannulation duration, min, M [IQR]	30.00 [25.00,30.00]	28.00 [22.00,40.00]	0.719	30.00 [24.00,30.00]	30.00 [20.00,40.00]	0.881
Insertion length, cm, M [IQR]	36.99 ± 6.05	37.35 ± 2.94	0.569	38.11 ± 6.03	37.31 ± 3.14	0.378
Exposure length, cm, M [IQR]	5.62 ± 1.52	5.30 ± 1.74	0.173	5.70 ± 1.40	5.32 ± 1.68	0.186
Catheter dwell time, days, M [IQR]	144.5 [109.75,183.5]	124.0 [68.00,151.25]	**0.006**	134.0 [90.5,174.5]	134.0 [63.5,156.5]	0.354

PSM = propensity score matching; BMI = body mass index; CHD = coronary heart disease; CRT = catheter-related thrombosis; PICC = peripherally inserted central catheter; PLT = platelet count; PT = ‌prothrombin time; APTT = ‌activated partial thromboplastin time; TT = ‌thrombin time; M = medium; IQR = interquartile ranges; SD = standard deviations.

### The comparative results for the primary observed outcomes

**Fig 2:**
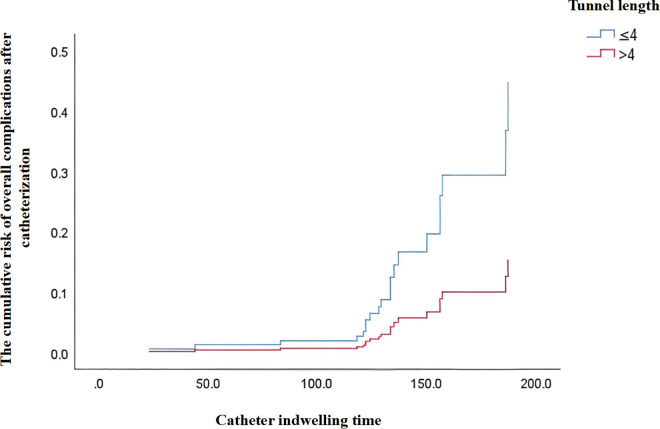
The cumulative risk of overall complications after catheterization.

To address baseline imbalances in cancer type, surgical history, and insertion vein type, and to account for potential confounding by catheter indwelling time, multivariate Cox regression was performed (**Table 2**). The risk of overall complications after catheterization in the observation group was 2.92 times that in the control group after adjustment[HR = 2.92, 95%CI: 1.07–7.94, *P* = 0.036; **Fig 2**]. No significant differences were observed in other complication outcomes, including blood oozing, MARSI, catheter dislodgement, local infection, and CRT (*P* > 0.05). Post-PSM analysis corroborated this finding.

**Table 2:** The primary outcomes of the participants. (N = 207).

Category	Groups	Before PSM					After PSM		
		n(%)	Before adjustment		After adjustment		n(%)	HR(95%CI)	*P* Value
			HR(95%CI)	*P* Value	HR(95%CI)	*P* Value			
Complications after catheterization	Control group	11 (8.2)	1	1	1	1	2 (3.5)	1	1
	Observation group	10 (13.7)	2.52 (1.03, 6.16)	**0.043**	2.92 (1.07, 7.94)	**0.036**	8 (14.0)	8.85 (1.06, 73.71)	**0.044**
Bleed oozing	Control group	5 (3.7)	1	1	1	1	0 (0.0)	1	1
	Observation group	4 (5.5)	2.07 (0.51, 8.34)	0.308	0.26 (0.02, 3.39)	0.305	3 (5.3)	—	0.448
MARSI	Control group	2 (1.5)	1	1	1	1	1 (1.8)	1	1
	Observation group	4 (5.5)	6.06 (1.07, 34.49)	**0.042**	4.83 (0.81, 28.67)	0.083	4 (7.0)	4.74 (0.49, 45.68)	0.179
Catheter dislodgement	Control group	0 (0.0)	1	1	1	1	0 (0.0)	1	1
	Observation group	1 (1.4)	—	0.581	—	0.581	1 (1.8)	—	0.592
Local infection	Control group	3 (2.2)	1	1	1	1	0 (0.0)	1	1
	Observation group	1 (1.4)	1.16 (0.12, 11.56)	0.899	0.74 (0.07, 7.86)	0.802	1 (1.8)	—	0.585
CRT	Control group	1 (0.7)	1	1	1	1	1 (1.8)	1	1
	Observation group	0 (0.0)	—	0.671	—	0.671	0 (0.0)	—	—

PSM = propensity score matching; MARSI = medical adhesive-related skin injury; CRT = catheter-related thrombosis.

### The comparative results for the secondary observed outcomes

**Table 3** compares the secondary outcomes of the two groups of participants. There was no significant difference in the patient’s comfort level between the two groups (*P* > 0.05), while the unplanned catheter removal rate was significantly higher in the observation group versus controls(4.4% vs. 0.0%, *P* = 0.027).

**Table 3:** Secondary outcomes of the participants (N = 207).

Category	Control group (n = 134)	Observation group (n = 73)	Z/χ2	*P* Value
Comfort levels.			0.013	0.909
Comfort	113 (84.3)	62 (84.9)		
Discomfort, tolerable	21 (15.7)	11 (15.1)		
Unplanned catheter removal rate	0 (0.0%)	4 (4.4%)	4.875	**0.027**

### Subgroup analysis

**Fig 3:**
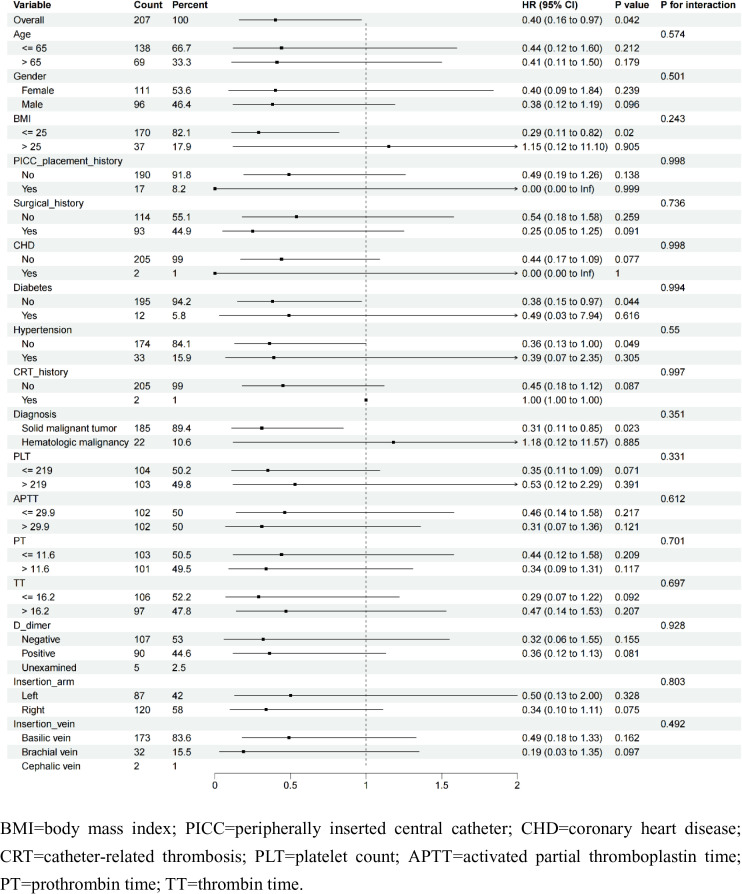
The subgroup analysis for the association between tunnel length and overall complications outcomes after catheterization.

Subgroup analyses were performed using a stratified Cox regression, with the observation group as control. All continuous variables except age and BMI were divided based on the median, with age at 65 years and BMI at 25 kg/m^2^ divided into two subgroups. The subgroup analysis showed that longer tunnel lengths (> 4 cm) were associated with a lower risk of overall complication after catheterization in patients with BMI ≤ 25 kg/m^2^ (HR = 0.29, 95% CI: 0.11–0.82), without hypertension (HR = 0.36, 95% CI: 0.13–1.00) or diabetes (HR = 0.38, 95% CI: 0.15–0.97), and with solid malignant tumor (HR = 0.31, 95% CI: 0.11–0.85), presented in **Fig 3**.

## Discussion

To our knowledge, this is the first multi-center retrospective study to compare clinical outcomes of tunneled PICCs with different tunnel lengths. The primary finding demonstrates significantly higher overall complication rates and unplanned catheter removal rates in patients with tunnel lengths ≤ 4 cm versus those with tunnel lengths>4 cm. Notably, longer tunnels (> 4 cm) were associated with prolonged catheter indwelling time, which may potentially contribute to the observed risk reduction. For patients with poor vascular conditions requiring tunneled PICCs, creating subcutaneous tunnels exceeding 4 cm is recommended to optimize exit site positioning and reduce complication risk.

Significant baseline differences in cancer type, surgical history, and insertion vein reflected the the inherent limitation of non-randomized assignment. The group size imbalance reflects real-world clinical decision-making patterns in this multi-center study, where clinicians preferentially use longer tunnels for complex cases. To mitigate selection bias, we employed PSM for baseline balance and multivariate Cox regression for confounder adjustment. This methodology improves upon previous single-center studies by incorporating both design-based and model-based adjustments [15].

The most important finding of this study was that the overall complication incidence after catheterization of the observation group (tunnel length ≤ 4 cm) was significantly higher than the other group (tunnel length >4 cm), which is similar to the results of previous studies [15]. The reason for the high incidence of overall complications after catheterization in patients with tunnel length ≤ 4 cm may be related to the fact that the tunnel length is too short to exert the advantages of subcutaneous tunnel in reducing complications, resulting in an effect close to that of conventional PICC. While extended tunnels may mitigate catheter movement, reducing mechanical irritation and microbial migration at the exit site—critical factors in thrombosis and infection pathogenesis. However, there was no significant difference in blood oozing and catheter dislodgement between the two groups with different tunnel lengths, which was inconsistent with the results of previous studies [15]. The failure to detect between-group differences in the incidence of bleed oozing and catheter dislodgement may be explained by the large sample size difference between the two groups. In addition, there was no significant difference between the two groups in terms of local infection, catheter-related thrombosis, and other complications after catheterization, which is similar to the finding of Li et al. [15].

In addition, this study also demonstrated that the tunnel length ≤ 4 cm had a higher unplanned catheter removal rate than the other group, aligning with Li et al. [15]. This likely reflects enhanced catheter fixation in extended tunnels, reducing the catheter removal incidence. However, there was no significant difference between the two groups regarding patient comfort level, confirming the previous finding [15].

The subgroup analyses revealed important differential effects based on patient characteristics, providing new insights into personalized catheter management. Specifically, longer tunnel (> 4 cm) demonstrated enhanced protective effects in patients with BMI ≤ 25 kg/m². This may be related to the fact that patients with BMI > 25 kg/m² need to build a longer tunnel due to thicker subcutaneous fat, so that tunnel length > 4 cm does not show more advantages in this population. The reduced efficacy of longer tunnels in hypertensive and diabetic patients may stem from impaired tissue repair mechanisms, as hyperglycemia exacerbates inflammation [23] and hypertension damages vascular endothelium [24]. Notably, the significant risk reduction in solid tumor patients versus hematologic malignancies may reflect differential inflammatory states, as elevated interleukin-6 levels in hematologic malignancies are known to promote endothelial dysfunction, which further supported the results of Wang et al. [25].

### Limitations

This study has several limitations. First, residual confounding persists due to group imbalance and observational design despite statistical adjustments. Second, while a multi-center design enhances generalizability, the moderate sample size precluded analysis of rare complications like CLABSI. Third, this study exclusively used single-lumen 4Fr PICCs; insufficient data on multi-lumen catheters (e.g., double-lumen) prevented conclusions about their suitability for longer tunnels, thus limiting the generalizability of our findings to clinical scenarios involving multi-lumen devices. Furthermore, the retrospective design limited causal inference, and the single geographic region (Hubei Province) may affect generalizability. Finally, short-term complication assessment without uniform follow-up to catheter removal may have missed late events. Future multi-center randomized trials with larger cohorts, extended monitoring until catheter removal, and diverse populations (including evaluations of multi-lumen catheters) are recommended. Despite these constraints, longer tunneled PICCs (> 4 cm) demonstrate superior safety and efficacy, which has great implications for clinical practice.

### Implications for policy and clinical practice

This study confirmed that tunneled PICCs with > 4 cm subcutaneous tunnels demonstrate superior safety and efficacy in adult cancer patients, prolonging catheter indwelling time and reducing overall complication risk despite some nonsignificant differences in specific outcomes. These findings support guideline revisions establishing 4 cm as the minimum recommended tunnel length, particularly for patients with BMI ≤ 25 kg/m², without hypertension/diabetes, and with solid malignancies. Implementation should prioritize standardized clinician training while addressing anatomical variations.

## Conclusion

This study confirmed that tunnel puncture techniques with a tunnel length of >4 cm are safer and more effective. Although some differences in complications were not detected, tunneled PICC with a tunnel length of > 4 cm had a lower risk of overall complications after catheterization, and catheter indwelling time was prolonged. Clinicians should prioritize tunnel lengths > 4 cm in patients with BMI ≤ 25 kg/m², without hypertension/diabetes, and with solid tumors, while considering individualized adjustments according to patients’ characteristics.

## Supporting information

S1 File Underlying data set. (XLSX)
